# Supplementary material for: Identification of photoperiod-induced specific miRNAs in the adrenal glands of Sunite sheep (Ovis aries)
Source: Front Vet Sci. 2022 Jul 22;9:888207. doi: 10.3389/fvets.2022.888207 (PMC9354845; doi:10.3389/fvets.2022.888207)
Supplement: Supplementary file 1 [file Data_Sheet_1.ZIP › Supplementary file/miRDeep2 file.zip!/miRDeep2 file/Novel_Structure/SPLP42A2/Novel_28.pdf]

Provisional ID	: NC_019471.2_28331
Score total	: 2.4
Score for star read(s)	: -1.3
Score for read counts	: 0
Score for mfe	: 2.1
Score for randfold	: 1.6
Score for cons. seed	:
Total read count	: 42
Mature read count	: 38
Loop read count	: 0
Star read count	: 4

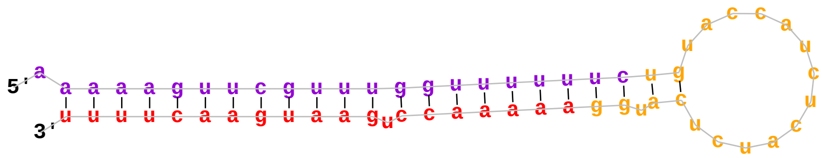

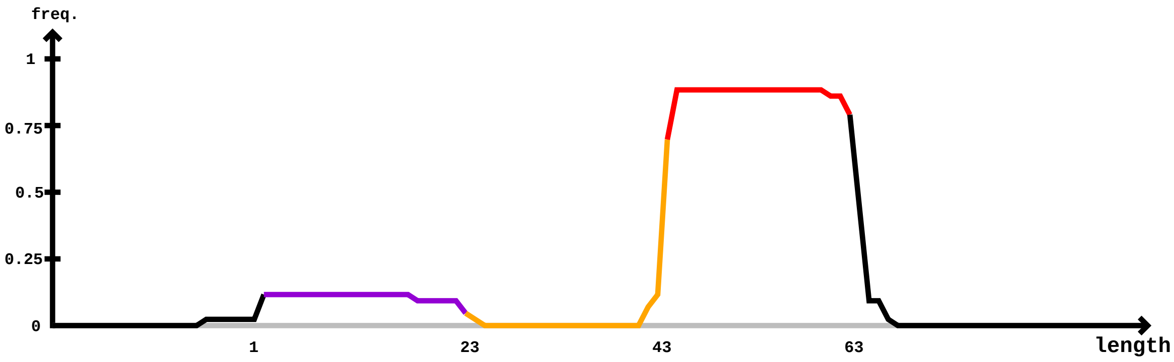


```
                         Star                                        Mature
5'- acuucuguauuggguuggcuaaaaaguucguuugguuuuuucuguaccaucucaucucauggaaaaaccugaaugaacuuuuggccaacccagcagaaauuaucaaggagga  -3'   obs
    acuucuguauuggguuggcuaaaaaguucguuugguuuuuucuguaccaucucaucucauggaaaaaccugaaugaacuuuuggccaacccagcagaaauuaucaaggagga         exp
    ..((((((..((((((((((((((.(((((((((((((((((((.............)).))))))))).))))))))))))))))))))))))))))..............   reads    mm     sample
    ..............uuggcuaaaaaguucguuuggu............................................................................   1        0      seq
    ....................aaaaaguucguuugguuuuuu.......................................................................   2        0      seq
    ....................aaaaaguucguuugguuuuuuc......................................................................   2        0      seq
    ............................................................ggaaaaaccugaaugaacuuu...............................   1        0      seq
    ............................................................Ugaaaaaccugaaugaacuuuu..............................   1        1      seq
    ............................................................ggaaaaaccugaaugaacuuuu..............................   1        0      seq
    .............................................................gaaaaaccugaaugaacuuC...............................   1        1      seq
    .............................................................gaaaaaccAgaaugaacuuuu..............................   1        1      seq
    ..............................................................aaaaaccugaaugaaUu.................................   1        1      seq
    ..............................................................aaaaaccugaaGgaacuuuu..............................   1        1      seq
    ..............................................................aaaaaccAgaaugaacuuuu..............................   10       1      seq
    ..............................................................aaaaaccugaaugaacuuuuC.............................   1        1      seq
    ..............................................................aaaaaccCgaaugaacuuuug.............................   10       1      seq
    ..............................................................aaaaaccugaaugaacuuuug.............................   1        0      seq
    ..............................................................aaaaaccugaaugaacuuuuUgcc..........................   1        1      seq
    ...............................................................aaaaccAgaaugaacuuu...............................   1        1      seq
    ...............................................................aaaaccAgaaugaacuuuu..............................   1        1      seq
    ...............................................................aaaaccCgaaugaacuuuug.............................   2        1      seq
    ...............................................................aaaaccugaaugaacuuuug.............................   1        0      seq
    ...............................................................aaaaUcugaaugaacuuuuggc...........................   3        1      seq
```
